# The relationship between congenital infections and autism spectrum disorder: a systematic review

**DOI:** 10.1016/j.jped.2026.101591

**Published:** 2026-08-11

**Authors:** Alice W. Jung, Júlia Supptitz, Pedro Nunes Hummes, Rafaela Jung Kurtz Rodrigues, Magda Lahorgue Nunes

**Affiliations:** a Pontifícia Universidade Católica do Rio Grande do Sul (PUC-RS), Escola de Medicina, Porto Alegre, RS, Brazil; b Instituto do Cérebro (InsCer), Pontifícia Universidade Católica do Rio Grande do Sul (PUC-RS), Porto Alegre, RS, Brazil

**Keywords:** Congenital infection, Autism spectrum disorder, Torch group, Neurodevelopmental disorders, Prenatal infections

## Abstract

**Objective:**

The aim of this systematic review (SR) is to evaluate the relationship between congenital infections (Toxoplasmosis, Cytomegalovirus, Rubella, Herpes simplex type 1 and 2, HIV, Zika, and Syphilis) and the development of Autism Spectrum Disorder (ASD).

**Data source:**

The authors seek to identify loopholes in the current knowledge about this content and to understand the role of congenital infections in children’s neurodevelopment. After the systematic search, 32 articles were included. Quality of articles was evaluated by the e Newcastle-Ottawa Scale (NOS).

**Findings:**

The data obtained were heterogeneous; the NOS varied from 4 to 9. In 19 studies, an association between congenital infection and the development of ASD and/or features of this spectrum was not observed. Furthermore, the present findings indicate that the link between congenital infections and ASD varies depending on the pathogen and there is no common causal factor among the diseases, as their mechanisms are not yet fully understood.

**Conclusion:**

This review highlights that there is a possible correlation between some congenital infections and the development of ASD, as is the case with CMV, Zika, Rubella and Toxoplasmosis infection. As the mechanisms are not yet fully understood, there is a need for further studies and research on this topic to bridge the existing knowledge gap regarding its mechanisms.

## Introduction

Autism Spectrum Disorder (ASD) was described by Leo Kanner in 1943. According to DSM-5, ASD is a neurodevelopmental disorder in which there are persistent deficits in communication and social interaction, associated with restricted and repetitive patterns in behavior, interests, and activities [1]. Moreover, ASD encompasses a broad, heterogeneous manifestation that can be influenced by environmental and genetic factors. It is believed that the environment could be associated with prenatal and postnatal interactions, and in this review, we’ll focus on this subject [2].

It is known that infection and inflammation during pregnancy can have an important impact on fetal brain development, although the mechanisms of this harm are not fully clarified. Although TORCH infections (Toxoplasma, Rubella, Cytomegalovirus, Herpes simplex, and Syphilis) pathogens are directly related to teratogenicity, emerging studies suggest their associations with a wider spectrum of lesions to fetal neurodevelopment. Over the past thirty years, more evidence has been raised, which indicates that a wide range of infections during pregnancy could lead to a higher risk of neurodevelopmental disorders, such as ASD [3].

Thus, the aim of this systematic review (SR) is to study if there is a relationship between congenital infections (Toxoplasmosis, Cytomegalovirus, Rubella, Herpes simplex type 1 and 2, HIV, Zika and Syphilis) and ASD. Above all, the authors seek to identify loopholes in the current knowledge about this content and to understand the role of congenital infections in children’s development. Therefore, the authors hypothesize that environmental interaction between an infectious pathogen with a fetal organism in development, associated with a genetic susceptibility, can lead to neurodevelopmental disturbance, especially ASD.

## Methods

This study is a systematic review. Hence, the following Mesh terms were used on databases from PubMED, Lilacs, and Embase, such as the following synonyms and acronyms: “Congenital Infection, *Toxoplasma gondii*” OR “CMV Infection, Congenital” OR “Rubella Syndrome OR “Congenital” OR “Congenital Zika Syndrome” OR “Virus, AIDS” OR “Syphilis, Congenital” AND “Autism Spectrum Disorders”. The search strategy was developed by an institutional librarian, reviewed by the senior researcher (MLN) and is available as supplementary material (Supplementary Table 1).

The inclusion criteria were children up to 7 years old with a diagnosis of congenital infection and confirmed ASD; articles written in English and published between 2014 - 2024, with the following designs (retrospective and prospective cohorts, longitudinal, cross-sectional, observational studies, case-control or case report). The exclusion criteria were articles in which the data presented did not allow ensuring how the diagnosis of congenital infection or ASD was based. This study was registered at PROSPERO (https://www.crd.york.ac.uk/prospero/) (ID CRD42024600301).

A total of four reviewers conducted the reading and selection of articles. The process was carried out in pairs, with each pair responsible for evaluating the inclusion and exclusion criteria. All articles retrieved from database searches were imported into Rayyan (https://rayyan.qcri.org), where duplicate articles were identified by the software and excluded after confirming titles and authors. Subsequently, the articles were screened by the four reviewers based on their title and abstract, with articles that did not address the relationship between one of the studied congenital infections and the development of ASD being excluded. After completing this stage, the reviewers performed a full-text reading of all selected studies for data extraction, which was carried out independently by the two pairs of reviewers.

The methodological quality of the included studies was assessed using the Newcastle-Ottawa Scale. Studies were classified according to their total score as follows: high quality, 7 to 9 points; moderate quality, 4 to 6 points; and low quality, 0 to 3 points. For analytical purposes, studies scoring 7 points or higher were considered to have good methodological quality, whereas studies scoring below 7 points were considered to have moderate or low methodological quality.

The extracted data were compiled into an Excel table with the following criteria: article characteristics (author identification, year of publication, journal name, DOI, title, objective, and study design) and sample characteristics (sample size, country of origin, follow-up period, and results). Discrepancies regarding the inclusion or exclusion of studies during this process were resolved through consensus discussions with a senior reviewer. To ensure the quality control of the selected articles, the Newcastle-Ottawa Scale (NOS) was applied, with scores ranging from 0 to 9, for case-control and cohort studies.

**Fig. 1:**
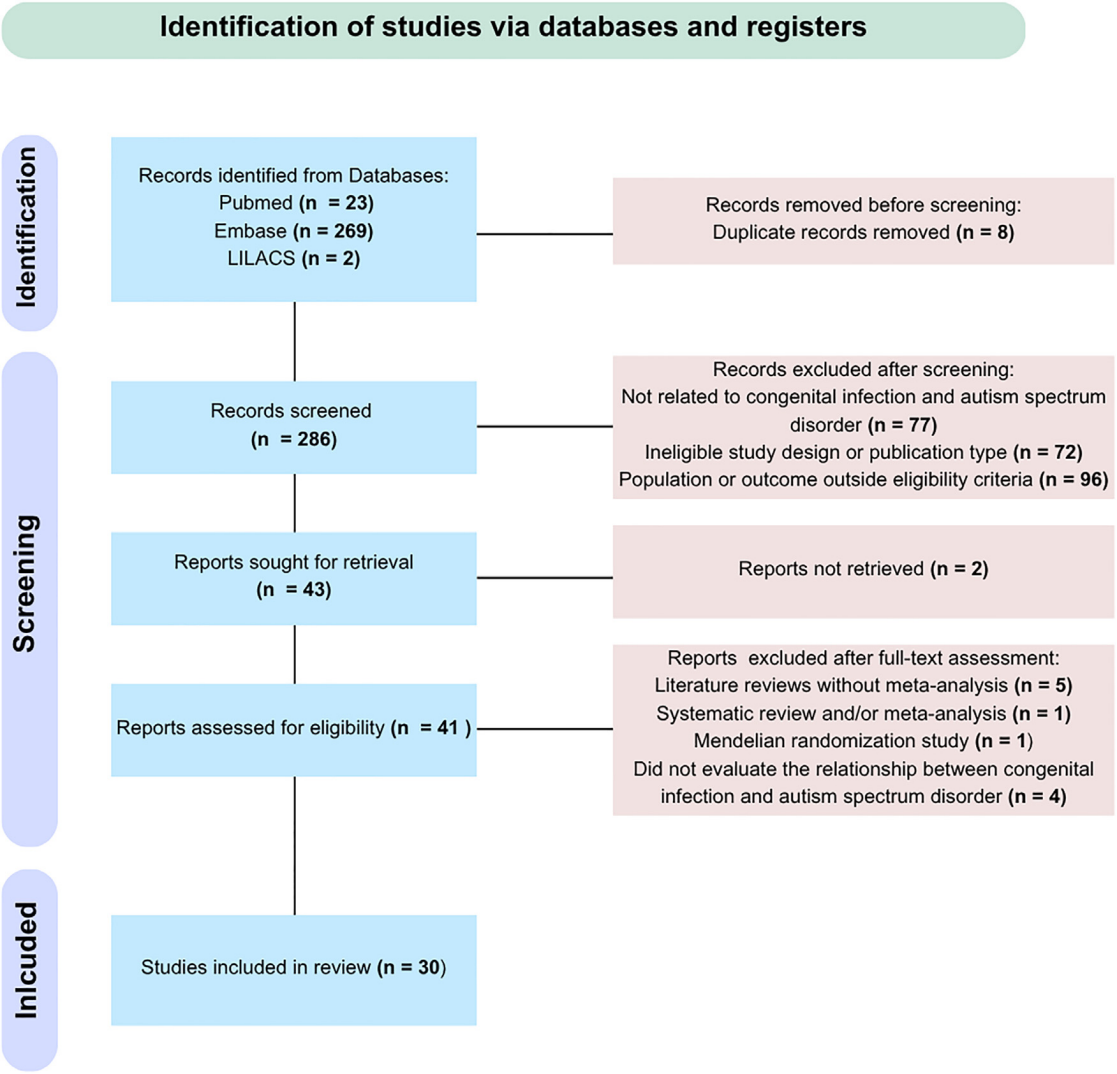
PRISMA flow chart showing the process of systematic article search and selection.

The present search yielded initially 286 articles, and after applying the inclusion criteria and performing a full-text review, 30 articles were included. To document the literature search process, the authors followed the Preferred Reporting Items for Systematic Reviews and Meta-Analyses (PRISMA) guidelines. Figure 1 presents the flow diagram outlining the study selection process.

## Results

Table 1 summarizes the data extracted from the 30 articles included in this review. Results are presented according to the type of congenital infection. Of the included studies, 14 were case-control studies (mean NOS score = 6.7), 5 were prospective cohort studies (mean NOS score = 7.0), 7 were retrospective cohort studies (mean NOS score = 6.0), 3 were cross sectional and 1 case report.

**Table 1:** Data description of included articles.

Title and Authors	Publication Year	Type of Congenital Infection	Study Design	NOS	n (Number of patIents)	Diagnostic criteria for TORSCH	Diagnostic criteria for ASD	Main findings
ENGMAN, M-L et al. *Acta Paediatrica*	2015	Cytomegalovirus	Retropective cohort	5	6.175	PCR	DSM-IV	Congenital CMV infection was identified in 1 of 33 children with ASD and ID, corresponding to 3% of this subgroup. No cases were found among 82 children with ASD without intellectual disability. The prevalence was higher than the reported prevalence of congenital CMV infection among newborns in Sweden, which was 0.2%.
SAKAMOTO, A. et al. *Brain & Development*	2015	Cytomegalovirus	Retropective cohort	6	3257	PCR	DSM-IV, DSM-V, M-CHAT, CARS.	Although two cases of ASD were identified among 27 children with CMV infection, the study could not establish a definitive causal link.
INABA, Y. et al. *Pediatric Neurology*	2016	Cytomegalovirus	Cross-sectional study	-	9	PCR (Umbilical cord)	DSM-V, Wechsler Intelligence Scale for Children and Brain MRI	No associations were found between CMV infection and ASD.
GAROFOLI, F. et al. *Journal of Autism and Developmental Disorders,*	2017	Cytomegalovirus	Retrospective Cohort	7	70	PCR and/or Shell (urine test)	DSM IV-TR	Congenital CMV infection may affect neurological development, potentially increasing the risk of autism.
GENTILE, I. et al. In Vivo	2017	Cytomegalovirus	Case control Study	7	82	PCR	DSM-IV, DSM-IV TR, DSM-V, ADOS, GMDS and VABS	The prevalence of congenital CMV infection was higher among children with ASD (5%) than in the general population (0.6%), difference was not statistically significant, findings only suggest a possible association.
SLAWINSKI, BL. et al. *American Journal of Reproductive Immunology*	2019	Cytomegalovirus	Prospective Cohort	9	82	Enzyme-linked immunosorbent assay (ELISA)	BAPQ and SRS2	Maternal seropositivity for CMV may be associated with higher levels of ASD symptoms in children.
LIN, C—H. et al. *Children*	2021	Cytomegalovirus	Retrospective Cohort	7	361	PCR	DSM-IV and DSM-V	The earlier CMV infection occurs infancy, the greater the risk of developing epilepsy and autism spectrum disorder later in life, particularly among children infected before 2 years of age.
NATSUME, T. et al. *Neuropediatrics*,	2022	Cytomegalovirus	Case control Study	9	34	PCR	DSM-V and brain MRI	A significantly higher proportion of children with CMV infection were diagnosed with ASD (47%) compared with the control group,
YANG, X-Y. et al. *Microbiology Spectrum*	2022	Cytomegalovirus	Case Control Study	7	401	PCR, CLIA and Western Blot	DSM-V	CMV infection contributed to the occurrence of ASD in TSC populations, which supported the theory of certain genetic ASD subpopulations having different sensibility to specific environmental risk factors
KEYMEULEN, A. et al. *Early Human Development*	2023	Cytomegalovirus	Prospective Cohort	5	753	PCR	AIMS, Bayley Scales of Infant Development (II/III edition), Wechsler Intelligence scales for children (III Edition)	ASD was diagnosed in 24.6% of children with behavioral problems, (2.5% of the total sample), the rate is higher than the reported prevalence in the general population of Flanders (0.7%), though suggesting a possible association.
GENTILE, I. et al. *In Vivo*	2014	HSV 1 and HSV 2	Case control Study	5	100	CLIA, and Specific IgG antibodies for HSV-1 and HSV-2	DSM-IV, CARS, ADOS-Generic, Clinical evaluation	The seroprevalence rate and total antibody levels were similar between individuals with autism spectrum disorder and age-matched healthy controls.
ZAPPULO, E. et al. *In Vivo*	2018	HSV-1 and HSV-2	Case control Study	7	82	PCR	DSM-IV, DSM-IV-TR, DSM-V, GMDSER, ADOS, ADI-R, CARS, VABS.	No causal relationship was found between HSV-1 and HSV-2 infection and autism spectrum disorder.
TOIZUMI, M. et al. *Scientific Reports*	2017	Rubella	Prospective Cohort	4	41	Serology (IgG and IgM)	ASQ-3, Denver II, M-CHAT, CARS-2, DSM-IV, DSM-V.	Among children with congenital rubella syndrome, 71% had global developmental delay and 41% screened positive for ASD., Authors noted that ASD assessment was difficult because many children had combined sensory impairments.
KADHIM, S J. et al. *Journal of Pharmaceutical Sciences and Research*,	2018	Rubella	Case Control Study	6	80	ELISA (IgG) and Combo Rapid Test Device (IgG and IgM)	DSM-V	The study did not find a significant association between maternal rubella infection during pregnancy and ASD. However, maternal seronegativity for rubella-specific IgG was discussed as a possible factor related to ASD risk, suggesting that lack of preexisting immunity, rather than infection itself, may have a role.
HUTTON, J et al. *Journal of Medical Virology*	2023	Rubella	Cross-sectional Study		296	Seroconversion in the mother	Self-assessment of parents of guardians of children	Autism was more frequent among children born to women who seroconverted for rubella during pregnancy than among those whose mothers remained nonimmune, with rates of 12.5% and 3.9%, respectively; however, the difference was not statistically significant.
GRETHER, J K. et al. *Frontiers in Neuroscience*	2016	Toxoplasmosis	Case Control Study	8	292	ELISA	DSM-V + Protocol developed by the Metropolitan Atlanta Developmental Disabilities Surveillance Program.	Higher levels of *Toxoplasma gondii* IgG in both maternal mid-gestational and newborn specimens were associated with lower risk of ASD. Overall, lower immunoglobulin levels tended to be associated with higher ASD risk, although most comparisons did not reach statistical significance.
SPANN, MN. et al. *Autism Research,*	2016	Toxoplasmosis	Case control Study	8	1748	CMIA + AVIcomp (IgG avidity to *Toxoplasma gondii*)	ADI-R	High maternal *T. gondii* IgM levels during pregnancy were associated with lower odds of childhood autism in offspring. In contrast, low-positive maternal *T. gondii* IgG levels were associated with higher odds of childhood autism. These findings suggest that the association may be related to maternal immune response rather than direct evidence of congenital infection.
ESNAFOGLU, E. et al. *The Journal of Psychiatry and Neurological Sciences*,	2017	Toxoplasmosis	Case Control Study	8	153	ELISA	DSM-V and CARS	3/102 children with ASD, were positive for IgG, whereas only one child in the control group showed IgG positivity. Difference not statistically significant.
*AL MALKI, J. S.* et al. *BMC Pediatrics,*	*2021*	*Toxoplasmosis*	*Case Control Study*	*4*	*108*	ELISA and PCR	ASD diagnostic criteria not exposed. All participants had previous diagnosis by specialists in government hospitals.	Toxoplasmosis was detected in 33% of samples by ELISA and in 80% by nested PCR. Mutations in mtDNA and nDNA genes were also reported among autistic children with toxoplasmosis, suggesting a possible link between *T. gondii* infection, genetic alterations, and ASD.
HAMID, N. et al. *The Pediatric Infectious Disease Journal*,	2022	Toxoplasmosis	Cross-sectional Study		100	ELISA	43 item questionnaires with Likert scale for assessing the physical, communicational, verbal aggression and impulsive anger of preschool children	*T. gondii* infection was significantly more frequent among children with ASD than among controls, with rates of 34% and 10%, respectively. Among children with ASD, 16% had acute infection and 18% had chronic infection, compared with 2% and 8% among controls. *T. gondii* infection was also associated with higher aggression scores in children with ASD.
EL-SAYED, SH. et al. *The Egyptian Journal of Neurology, Psychiatry and Neurosurgery*,	2024	Toxoplasmosis	Case Control Study	9	100	ELISA (IgG and IgM from blood)	DMS-V and Childhood Autism Rating Scale (CARS)	There is a relationship between ASD and IgG to Toxoplasmosis, however it does not entail worsened autistic features.
GENTILE, I. et al. *Future Virology*	2017(a)	Varicella Zoster Virus (VZV)	Case Control Study	6	82	Viral DNA detection and PCR	DSM-IV, DSM-IV-TR, DSM-V	Congenital infection with varicella-zoster virus does not appear to be a significant etiological factor in the development of ASD.
NIELSEN-SAINES, K. et al. *Nature Medicine*	2019	Zika	Prospective Cohort	8	216	PCR	Bayley-III Scales of Infant and Toddler Development, neurodevelopmental questionnaires and neurological exam	Three children who had been exposed to Zika virus during pregnancy were diagnosed with ASD during their second year of life.
GAZETA, RE. et al. *Viruses*	2021	Zika	Prospective Cohort	8	799	PCR	No description	Developmental abnormalities were more frequent among children exposed to or infected with ZIKV than among controls, with rates of 13.2% versus 3.8%. ASD was reported in 2.9% of children in the exposed/infected group.
GRANT, R. et al. *BMC Medicine*	2021	Zika	Prospective Cohort	9	235	PCR or IgG or IgM Anti-Zika Virus	ASQ, M-CHAT and IFDC	At 24 months of age, abnormal neurodevelopmental findings were observed in 15.3% of children exposed to Zika virus in utero.
SANTI, L. et al. *Neuroimmunomodulation*	2021	Zika	Case Report		1	ELISA and Clinical examination	DSM-V e ASQ	One child with positive ZIKV IgG and prenatal exposure had normal neonatal findings, but developed neurodevelopmental regression around 20 months of age and was later diagnosed with ASD.
GUARDADO, K. et al. *Children*	2022	Zika	Retrospective Study	4	47	Multiple criteria: Laboratory techniques), probable cases(any pregnant woman presenting two or more symptoms compatible with Zika and with a history of visiting or residing in endemic areas).	The Denver Developmental Screening Test II EID and DDST-II	Among children exposed to ZIKV during pregnancy, 72.2% had either risk of developmental delay or developmental delay by EDI, while DDST-II showed delays most frequently in fine-adaptive motor skills (88%).
ROTH, NM. et al. *Morbidity and Mortality Weekly Report (MMWR)*	2023	Zika	Retrospective cohort	6	3.122	PCR and Nucleic acid amplification testing (NAAT) (Detection of Zika virus RNA)	DSM-V	Suggest an association between Zika virus exposure and potential adverse neurodevelopmental outcomes, but causal effect was not conclusive.
HASSAN, ZR. et al. *Parasitol Research*	2023	Toxoplasmosis and Cytomegalovirus	Case Control Study	5	90	ELISA and RT-PCR	CARS	The findings did not support a statistically significant association between *T. gondii* infection or CMV infection and ASD.
LIN, C.-H. et al. *Frontiers in Neurology,*	2019	Enterovirus, Group B *Streptococcus, Streptococcus pneumoniae*, and Herpes Simplex Virus	Retrospective cohort	7	437	PCR and Positive CSF culture	DSM-IV and DSM-V	In the infected group, children aged 2 to 5 years showed a statistically significant association with ASD, with a probability value of 0.03.

Note: ADI-R, Autism Diagnostic Interview-Revised; ADOS, Autism Diagnostic Observation Schedule; AIMS, Alberta Infant Motor Scale; ASD, autism spectrum disorder; ASQ, Ages and Stages Questionnaire; ASQ-3, Ages and Stages Questionnaire, Third Edition; BAPQ, Broad Autism Phenotype Questionnaire; CARS, Childhood Autism Rating Scale; CARS-2, Childhood Autism Rating Scale, Second Edition; CLIA, chemiluminescent immunoassay; CMIA, chemiluminescent microparticle immunoassay; CMV, cytomegalovirus; CSF, cerebrospinal fluid; DDST-II, Denver Developmental Screening Test II; DSM-IV, Diagnostic and Statistical Manual of Mental Disorders, Fourth Edition; DSM-IV-TR, Diagnostic and Statistical Manual of Mental Disorders, Fourth Edition, Text Revision; DSM-5, Diagnostic and Statistical Manual of Mental Disorders, Fifth Edition; EID, Evaluation of Infant Development; ELISA, enzyme-linked immunosorbent assay; GMDS, Griffiths Mental Development Scales; HSV-1, herpes simplex virus type 1; HSV-2, herpes simplex virus type 2; IFDC, French MacArthur Communicative Development Inventories; IgG, immunoglobulin G; IgM, immunoglobulin M; M-CHAT, Modified Checklist for Autism in Toddlers; MRI, magnetic resonance imaging; NAAT, nucleic acid amplification testing; NOS, Newcastle-Ottawa Scale; PCR, polymerase chain reaction; RNA, ribonucleic acid; RT-PCR, reverse transcription polymerase chain reaction; SRS-2, Social Responsiveness Scale, Second Edition; *T. gondii, Toxoplasma gondii*; TORSCH, toxoplasmosis, other agents, rubella, syphilis, cytomegalovirus, and herpes simplex virus; TBS, tuberous sclerosis complex; VABS, Vineland Adaptive Behavior Scales; VZV, varicella-zoster virus.

## Cytomegalovirus

A total of 10 articles addressed whether there is a relationship between ASD and Cytomegalovirus (CMV) [[4], [5], [6], [7], [8], [9], [10], [11], [12], [13]]. One further study included CMV and toxoplasmosis patients [14]. These studies were published between 2015 and 2023. The design and quality of studies varied, and data was heterogeneous. Except for two studies that did not find a significant relationship between CMV and ASD, [13,14] the others suggested a higher prevalence of ASD in association of CMV congenital infection, some of them comparing the rates obtained to what was report regarding previous populational data [4,6,9].

## Zika

Six articles studied ASD and the congenital Zika virus infection [[15], [16], [17], [18], [19], [20]]. Two prospective and 2 retrospective cohorts, one case-control and one case report. The quality of studies varied, and data was heterogeneous.

Patients with Zika virus infection were at higher risk for developing neurodevelopmental abnormalities including ASD and its prevalence was higher than in the general population in some studies [15,16,20]. Also, more abnormalities in language, communication, fine and gross motor skills, cognitive and social-personal areas were reported [17,18]. In addition, there was a higher prevalence of male gender in the affected group [16]. Earlier exposition in pregnancy was associated with worse ASD features [17,18]. A case report supported that ASD can be a later manifestation of Zika virus infection even in children who do not have features of congenital Zika syndrome [19].

## Toxoplasmosis

Six articles studied the relationship between ASD and Toxoplasmosis, which were published between 2016 and 2024, and one included mixed CMV and toxoplasmosis [14]. Six articles were case-control studies, [14,[21], [22], [23], [24], [25]] whose NOS scale ranged from 4 to 9, with a mean score of 7. Also, there was one cross-sectional study [26]. One case-control study found a significantly higher prevalence of IgG antibodies against *T. gondii* in the ASD group [25] and one cross-sectional study reported a higher prevalence of *T. gondii* infection in ASD children compared to a comparison group (p < 0.001) [26]. However, no clear association was observed in the others [14,[21], [22], [23], [24]].

## Rubella

Three articles studied the relationship between ASD and Rubella [[27], [28], [29]] and were published between 2017 and 2023. A prospective cohort obtained an NOS score of 4 and a case-control of 6, while the last one was a cross-sectional study.

Patients with Congenital Rubella Syndrome had a higher risk for neurodevelopmental disability, particularly in communication and language domains [28] and children from mothers who seroconverted to rubella had a higher prevalence of an ASD outcome, however, the relationship did not achieve statistical significance [27].

In contrast, a small case-control study showed that individuals with seronegative of anti-rubella IgG may consider at risk for ASD while IgG seropositivity had a protective effect [29].

## Herpes

Two articles studied the relationship between ASD and Herpes. The studies were published in 2014 and 2018. One article was a case-control study [30] (NOS = 5) and another was a prospective cohort study (NOS = 7) [31]. None of them found a significant association between congenital herpes virus infection and ASD.

## Varicella

There was one article [32] that assessed whether there is a relationship between ASD and Varicella. The study was published in 2017, with a case-control design (NOS = 6). However, no significant association was identified.

## Other agents

There was one prospective cohort study (NOS = 7) that assessed the neurodevelopmental outcome in children who had Central Nervous System (CNS) infection by several pathogens and after evaluating the outcome for each causing agent Enterovirus appeared as another infectious agent associated to the development of ASD [33].

No articles regarding HIV and Syphilis were identified after applying the criteria for this review.

## Discussion

In this systematic review, the authors aimed to identify whether there is a relationship between congenital infections (Cytomegalovirus, Rubella, Herpes, Varicella, Zika, Toxoplasmosis, HIV, and Syphilis) and ASD. The data obtained were heterogeneous; however, 19 studies did find an association between congenital infection and the development of ASD and/or features of this spectrum. Furthermore, the present findings indicate that the link between congenital infections and ASD varies depending on the pathogen.

The majority of the studies suggest biological mechanisms of maternal immune activation, neuroinflammation, and genetic susceptibility as hypotheses that could explain a possible relationship between congenital infections and ASD. Infections during pregnancy trigger a strong maternal immune response, leading to an elevated level of pro-inflammatory cytokines that could affect the neurodevelopment of the fetus [26,33]. Epigenetic modifications were also addressed in some studies as possible mechanisms, suggesting that prenatal infections can generate long-term changes in gene expression related to synaptic function and neural connectivity. However, none of the studies were able to perfectly clarify the causality between infections and ASD, as well as the underlying mechanisms [[4], [5], [6], [7], [8], [9], [10], [11], [12],[15], [16], [17], [18], [19], [20],[24], [25], [26], [27], [28],^30^] Table 2 provides a summary of possible mechanisms that might explain the association of each congenital infection and the development of autism.

**Table 2:** Proposed pathophysiological mechanisms linking congenital infections to Autism Spectrum Disorder (ASD). .

Infectious agent	Proposed pathophysiological mechanisms associated with ASD
Cytomegalovirus (CMV)	Maternal immune activation and fetal neuroinflammation; increased production of pro-inflammatory cytokines and chemokines; direct injury to neural progenitor cells; disruption of neuronal migration and differentiation; impairment of neural circuit formation; microglial activation and altered synaptic pruning; white matter abnormalities; impaired brain connectivity; hippocampal alterations; interaction with genetic susceptibility.(4–13)
Zika virus (ZIKV)	Maternal immune activation and fetal inflammatory response mediated by pro inflammatory cytokines; disruption of neurodevelopmental processes; impairment of neuronal proliferation, migration, and maturation.(19)
*Toxoplasma gondii*	Maternal immune activation; mitochondrial DNA and nuclear DNA damage; oxidative stress and cellular injury; complex immunological interactions, with some evidence suggesting a protective role of robust maternal immune responses. (21,23–26)
Rubella virus	Impairment of retinol-to-retinoic acid conversion and retinoic acid catabolism; retinoid toxicity during foetal development; disruption of mbryonic neurodevelopmental pathways.(27–29)
Herpes simplex virus (HSV)	No specific mechanism established; insufficient evidence supporting a direct association with ASD. (30–34)
Varicella-zoster virus (VZV)	No pathophysiological mechanism identified; evidence currently insufficient. (32)
Congenital infections (general)	Maternal immune activation; elevated pro inflammatory cytokines; fetal neuroinflammation; epigenetic modifications affecting gene expression; altered synaptic function and neural connectivity; interaction between environmental and genetic factors. (4–12; 15–20; 24–30; 33)

ASD, Autism Spectrum Disorder; CMV, Cytomegalovirus; ZIKV, Zika virus; HSV, Herpes simplex virus; VZV, Varicella-zoster virus. (reference number).

Regarding CMV infection, the majority of the studies have shown a possible association between this congenital infection and an increased risk of ASD, since they demonstrated a higher prevalence of CMV infection among children with ASD compared to a comparison group [[4], [5], [6], [7], [8], [9], [10]]. This congenital infection is known to induce a pro-inflammatory state in the fetal brain, characterized by increased levels of cytokines and chemokines, which can disrupt normal neurodevelopmental processes. The included studies suggest mechanisms such as direct injury to neural progenitor cells, disruption of neuronal migration and differentiation, and impairment of neural circuit formation during critical periods of neurodevelopment [4,9]. Furthermore, infection-induced neuroinflammation, characterized by microglial activation and increased production of pro-inflammatory cytokines, may interfere with synaptic pruning and the maturation of neural networks involved in social communication and behavior [11]. Evidence of white matter abnormalities, impaired brain connectivity, and hippocampal alterations in children with congenital CMV infection further supports the hypothesis that these structural and functional changes may contribute to ASD-related phenotypes [12,13]. Overall, current evidence supports an association with increased risk, likely mediated by interactions among infectious, immunological, and genetic susceptibility factors [[5], [6], [7], [8],^10^]. In a systematic review and meta-analyses, Maeyama and collaborators found a high prevalence of congenital CMV infection in ASD cases (OR 11.31, 95% CI 3.07–41.66). However, all three included studies had serious limitations [34].

The literature reviewed that addressed the relationship between congenital infection by Zika virus was more emphatic in the association, with ASD as one of the developmental disorders identified. In one study, it was suggested that the inflammatory response mediated by pro-inflammatory cytokines, triggered by ZIKV during pregnancy, may contribute to the development of neurodevelopmental disorders, such as ASD [19].

The studies that analyzed congenital Toxoplasmosis raised conflicting results, however, some studies reported a higher prevalence of toxoplasmosis infection in children with ASD [25,26]. Serological and molecular analyses suggested a positive relationship, where maternal toxoplasmosis infection was linked to mitochondrial DNA (mtDNA) and nuclear DNA (nDNA) damage, which may play a relevant role in autism development [24]. On the other hand, 2 studies suggested an inverse association, pointing out that higher maternal and neonatal IgG antibody levels were associated with a reduced risk of ASD, [23] and positive maternal IgM antibodies linked to lower odds of autism [21]. IgM secretion is one of the immune system’s initial responses to protect against a pathogen, and one interpretation is that a sufficient immune response to acute maternal *T. gondii* infection during pregnancy may be protective against ASD risk in offspring [21]. Thus, there may be a more complex pathophysiological mechanism involved, which requires further investigations.

Findings concerning the association between congenital Rubella infection and ASD were heterogeneous [[27], [28], [29]] and maybe an explanation may be the varied methodologies used. However, there are enough pathophysiological mechanisms that might link ASD and congenital rubella infection. One of the strongest theories suggests that the Rubivirus impairs the enzyme responsible for the conversion of retinol to retinoic acid and the catabolism of retinoic acid, resulting in retinoid toxicity. This process could disrupt normal fetal development, increasing the risk of ASD. However, the study emphasizes that, with the currently available evidence, it is still not possible to determine whether ASD is part of Congenital Rubella Syndrome. Therefore, further research is essential to clarify the role of Rubella in ASD.

The scarce data obtained in this review that evaluated a possible association between Herpes virus infection and ASD did not reach statistical significance. This finding was supported by previous literature [35] and the direct relationship between Herpes virus and ASD remains uncertain.

No pieces of evidence for an association between Varicella Zoster and ASD were obtained in this review. As only one single article, [32] with a small sample size, was retrieved in this review, more studies on this subject are necessary.

Moreover, other pathogens than the TORCH group can lead to neurodevelopmental impairment and ASD, such as Enterovirus [33] and it ought to trigger research into other infectious agents also.

Data obtained in this review allowed us to conclude that there is plausible evidence of a possible association between CMV, Zika, Toxoplasmosis, and Rubella infections and the development of ASD. Considering that congenital infections can disrupt neurogenesis and ASD has a multifactorial etiology, this is not an unexpected finding. Although there is no certainty of a common causal factor among the diseases, as their pathogenic mechanisms are not yet fully understood, this review highlights the possible positive association between congenital infections and ASD development, but there is an urgent need for more detailed studies and research on this topic.

## Statements and declarations

Not applicable.

## Ethical approval

Not required.

## Consent statements

Not applicable.

## Funding statement

This work did not receive any specific financial support. MLN is a 1D researcher supported by the National Council for Scientific and Technological Development (CNPq)- Brazil, PQ 303,168/2021–8. RJKR and AWJ shared the PUCRS (BPA program) and CNPQ scholarship to develop this study.

## Conflicts of interest

The authors declare no conflicts of interest.

## Data Availability

The data that support the findings of this study are available from the corresponding authors upon reasonable request, if this research is accepted for publication.

## Appendix Supplementary materials

Image, application 1

## Footnotes

Supplementary material associated with this article can be found in the online version at doi:10.1016/j.jped.2026.101591.
