# Supplementary material for: The relationship between congenital infections and autism spectrum disorder: a systematic review
Source: J Pediatr (Rio J). 2026 Aug 11;102(5):101591. doi: 10.1016/j.jped.2026.101591 (PMC13487289; doi:10.1016/j.jped.2026.101591)
Supplement: Supplementary file 1 [file mmc1.docx]

**JPED-D-26-00071_**Supplemtary Table 1

Search strategy
